# Divergent Abiotic Stressors Drive Grassland Community Assembly of Tibet and Mongolia Plateau

**DOI:** 10.3389/fpls.2021.715730

**Published:** 2022-01-03

**Authors:** Jianming Wang, Mingxu Li, Li Xu, Congcong Liu, Pu Yan, Nianpeng He

**Affiliations:** ^1^Key Laboratory of Ecosystem Network Observation and Modeling, Institute of Geographic Sciences and Natural Resources Research, Chinese Academy of Sciences, Beijing, China; ^2^College of Resources and Environment, University of Chinese Academy of Sciences, Beijing, China; ^3^Key Laboratory of Vegetation Ecology, Ministry of Education, Northeast Normal University, Changchun, China

**Keywords:** trait, community assembly, abiotic filtering, weaker competitive exclusion, stress, climate change

## Abstract

Multiple ecological processes simultaneously govern community assembly, but it remains unclear how abiotic stressors regulate the relative importance of these processes among different biogeographic regions. Therefore, we conducted a comprehensive study on the responses of community assembly to varying environmental gradients, using the mean, variance, skewness, and kurtosis of plant height (height), specific leaf area (SLA) and leaf dry matter content (LDMC) distributions on the Tibetan Plateau (TP) and the Mongolian Plateau (MP). Our results showed that the prevalence of trait convergence across all grasslands in both TP and MP seem to be the result of abiotic filtering or weaker competitive exclusion etc. These trait-convergence assembly processes decrease the functional dispersion but increase the evenness of the trait frequency distribution. The mean, variance, skewness, and kurtosis responses of grassland communities to abiotic stress varied between the TP and MP. On average, plant trait distribution was mainly driven by temperature on the TP, and low-temperature stress altered the community assembly rules. In contrast, water availability shaped plant trait frequency distributions on the MP, and drought stress mediated the balance between different assembly processes. Our results provide empirical evidence that divergent abiotic stressors regulate the grassland community assembly on the TP and MP. Together, our study speculates that different aspects of future climate change, such as climate warming and changing precipitation patterns, on community assembly are dependent on regional climatic regimes.

## Introduction

Elucidating the mechanisms and drivers of plant community assembly is a key challenge in ecology (Keddy, 1992; Vellend, 2010; Yao et al., 2021). However, many studies suggest that the traditional taxon-based approach is unable to adequately describe the influence of climate on plant community assembly processes (McGill et al., 2006; Götzenberger et al., 2012; Purschke et al., 2013; Cadotte and Tucker, 2017). Functional traits characterize the ecological strategies that species use to respond to environmental variations (Dìaz and Cabido, 2001; Violle et al., 2007). In nature, plant communities along environmental gradients exhibit distinct functional trait frequency distributions (Wieczynski et al., 2019; Liu et al., 2020), and the shifts in trait distributions are linked to community assembly processes and their responses to climate change (Enquist et al., 2015). Neutral theory posits that all individuals in a community are ecologically equivalent, and stochastic processes produce a random local trait frequency distribution (Cadotte and Tucker, 2017; Perronne et al., 2017). In contrast, a non-random trait frequency distribution pattern is observed if deterministic processes dominate (Kraft and Ackerly, 2010). Recent studies have suggested that multiple processes regulate community assembly, and changes in the relative strength of these processes may induce substantial shifts in trait distributions (Enquist et al., 2015). Therefore, exploring the influence of the underlying processes on trait distribution may provide new insights into community assembly mechanisms.

Abiotic filtering and biotic interactions have long been recognized as the predominant forces of community assembly (Emerson and Gillespie, 2008; Swenson et al., 2012). Abiotic filtering tend to preferentially select species with specific traits enter into community, resulting in coexistence of functionally similar species (i.e., trait convergence). Limiting similarity holds that biotic interactions, such as competition and parasitism, may prevent species from being too similar, leading to the niche differentiation of coexisting species possessing dissimilar traits (Macarthur and Levins, 1967; Cornwell and Ackerly, 2009; Bernard-Verdier et al., 2012). However, weaker competitor hypothesis believes that competitive interaction also leads to trait convergence patterns, because species bearing traits associated with low competitive ability are likely to be excluded by highly competitive species sharing traits conferring higher fitness, resulting the coexistence of functionally similar species (Grime, 2006; Mayfield and Levine, 2010). Additionally, facilitative interactions have been found to result in trait convergence among existing species (Moeller, 2004). Both the importance of abiotic filtering and biotic interactions has been demonstrated across different scales and ecosystems (Ding et al., 2019; Fang et al., 2019; Luo et al., 2021). However, their relative roles might be shaped by abiotic stressors (Lhotsky et al., 2016; Luo et al., 2019). Bernard-Verdier et al. (2012) found that resource availability mediated assembly rules in a Mediterranean rangeland. Furthermore, different abiotic stressors may influence species strategies differently. For example, resource availability is the main driver of different strategies along the leaf economic spectrum under high precipitation, whereas hydraulic constraints prevail under arid conditions (Blanchard et al., 2019). Therefore, testing the effect of multiple abiotic stressors on trait distribution provides a great opportunity to understand and predict the response of community assembly to climate change (Le Bagousse-Pinguet et al., 2017). Nevertheless, how multiple abiotic stressors jointly drive variations in the distribution of traits within communities across different spatial scales remains unclear.

Large-scale tests of trait–environment relationships have focused on trait distributions that span multiple biomes and biogeographic regions (Bruelheide et al., 2018). However, each region often has unique historical and biogeographic features (such as species pool, soil, and climate) (Zhang et al., 2016). These complex differences in historical and biogeographic conditions among different regions may be confounded in testing local community assembly processes (Ricklefs and He, 2016). As the direction and rates of trait evolution, dispersal, and speciation differ among biogeographic regions (Weir and Schluter, 2007; Cooper and Purvis, 2010), the manner in which species strategies respond to abiotic stressors may depend on the environmental regime (Muscarella et al., 2016; Crous et al., 2018). For example, water availability generally determines biodiversity in regions with high energy inputs, whereas temperature is more important for cold areas or higher altitudes (Hawkins et al., 2003; Kreft and Jetz, 2007). However, few studies have focused on elucidating how trait frequency distributions in different regions with different limiting factors are affected by abiotic stressors.

**FIGURE 1:**
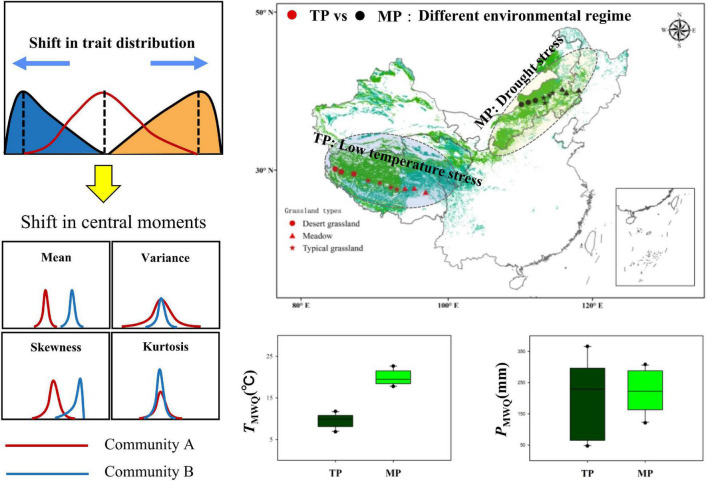
Two comparative grassland transects on the Tibetan Plateau (TP) and the Mongolian Plateau (MP). They represent different environmental regimes. Each transect covers meadows, typical grasslands, and desert grasslands. The shifts in central moments of trait distribution represent the difference in intrinsic structure between two communities.

To explore how multiple abiotic stressors affect plant community assembly among regions, regional-scale transect surveys were conducted across major grassland types within two representative Eurasian grassland biogeographic regions (Figure 1). The Tibetan Plateau (TP), referred to as the “the world’s roof,” covers the highest alpine grasslands, which experience low temperatures as a result of their altitude (Liu et al., 2018). Recently, the rate of climate warming on the TP has been more than twice the global average (Ma et al., 2017; Yao, 2019). The Mongolian Plateau (MP) covers the largest regions of Eurasian temperate grasslands, which are characterized by extremely limited water and nutrient availability (Wang J. et al., 2021). MP is also expanding owing to climate warming and frequent extreme weather events (Easterling et al., 2000; Dai, 2013). Both TP and MP have continuous natural vegetation gradients, which range from desert grassland to typical grassland to meadow, and similar precipitation gradients from west to east, making them ideal systems for conducting biogeographic comparisons of the responses of community assembly to climate change.

Here, the frequency distribution of three key functional traits of 80 grassland communities was quantified using the four important moments (i.e., mean, variance, skewness, and kurtosis) in each region (Figure 1). We specifically address the following questions: (1) What is the relative importance of stochastic and deterministic processes in these two regions? (2) Does the response of trait frequency distribution to abiotic stressors differ between the two regions? (3) What are the foremost abiotic stressors regulating community assembly processes in two regions?

## Materials and Methods

### Study Regions

In 2018, field investigations were conducted across the alpine and temperate regions of the eastern part of the Eurasian grasslands, including the TP and MP (Figure 1). In each region, an east–west transect was established, ranging from arid to mesic grasslands with varied soil, climatic, and vegetation conditions. The transect on the TP includes three dominant vegetation types (desert grassland, typical grassland, and meadow) with decreasing mean annual temperature (MAT, ca. 0.8 to –2.96°C) and increasing mean annual precipitation (MAP, ca. 75–606 mm) from desert grassland to meadows. On the TP, desert grasslands were dominated by *Stipa tianschanica var. gobica* and *Ajania fruticulose*, typical grasslands were dominated by *Stipa purpurea* and *Stipa capillata*, and meadows were dominated by *Kobresia pygmaea* and *Potentilla saundersiana*, among others. The transect on the inner MP includes three dominant vegetation types (desert grassland, typical grassland, and meadow) with increasing MAP (ca. 183–425 mm) from desert grassland to meadows, whereas average MAT was the highest in meadows (ca. 3.91–6.64°C), followed by desert grasslands (ca. 1.81–3.81°C), and the lowest in typical grasslands (ca. 0.98–1.59°C). On the MP, desert grasslands were dominated by *Stipa breviflora* and *Stipa klemenzii;* typical grasslands were dominated by *Stipa grandis* and *Artemisia frigida*, and meadows were dominated by *Stipa baicalensis* and *Leymus chinensis*. The Wilcoxon test showed that the soil and climatic conditions were significantly different between the two grassland transects (Figure 1 and Supplementary Figure 1). Together, these transects were ideal systems for examining the macroscale drivers of community assembly.

### Field Sampling

At each east–west transect, 10 survey sites were randomly selected along the vegetation gradient, including three for desert grassland, four for typical grassland, and three for meadow. At each site, eight 1 m × 1 m quadrats were randomly established within a 1 km × 1 km sampling area, and the geographic coordinates and elevations of each quadrat were recorded using a Global Positioning System (GPS) device. In total, 24 quadrats of desert grassland, 32 quadrats of typical grassland, and 24 quadrats of meadow were selected in each biogeographic region. For each quadrat, all plant species and their individuals were identified, and the plant coverage of each species was visually estimated. The height of each species was determined by measuring the height of 50 randomly selected individuals from each site. Approximately 50 mature but non-senescent leaves with little damage were collected from different locations at each site (outside of each quadrat) to determine leaf dry matter content (LDMC) and SLA. Plant samples were detached to measure plant height, SLA, and LDMC using a previously described procedure (Cornelissen et al., 2003; Pérez-Harguindeguy et al., 2013). The “species mean” trait values were calculated by averaging the trait values of all repetitions of a given species sampled across 10 sites. Aboveground biomass was clipped by species in each 1 m × 1 m quadrats, and dead parts were removed and combined with plant litter. The leaf and aboveground biomass samples were carefully cleaned and oven-dried at 60°C. The aboveground biomass of each species within each quadrat was measured. According to soil heterogeneity, 20–30 soil cores (10 cm in depth) were randomly collected in each quadrat and subsequently mixed into a composite sample.

### Soil and Climate Data

All soil samples were air-dried after being sieved (2 mm mesh), and visible roots and organic debris were removed. We summarized soil parameters at each quadrat using soil total nitrogen (*S*_*TN*_) and phosphorus (*S*_*TP*_) content, and soil pH (*S*_*pH*_). *S*_*pH*_ was determined using a 1:2.5 (v/v) soil water aqueous extract. The *S*_*TN*_ and *S*_*TP*_ contents have been calculated in a previous study (Zhang et al., 2020).

As climate variables, the mean temperature of the warmest quarter (*T_*MWQ*_*) and temperature seasonality (*T*_*S*_) were selected to represent the temperature stress; the precipitation of the wettest quarter (*P*_*MWQ*_), and precipitation seasonality (*P*_*S*_) were selected to represent the water stress. *T*_*S*_ indicates the difference between the annual maximum and minimum temperatures, whereas *P*_*S*_ reflects the differences in the seasonal distribution of precipitation between locations in the form of alternating dry and wet seasons. These climatic data were obtained from the WorldClim global climate database using geographic coordinates for each site (with a resolution of 1 km × 1 km).^1^

### Trait Frequency Distribution

Three key plant traits, namely, height, SLA, and LDMC, were used in this study. Plant height is a central trait for plant ecological strategies and is strongly correlated with seed mass, life span, and time to maturity (Zhang et al., 2016). Height is key to a species’ carbon gain strategy because it is a major determinant of the species’ competitive ability to light (Díaz et al., 2016). SLA describes the amount of leaf area for light capture per unit of biomass invested, which reflects the trade-offs between leaf structural attributes, carbon gain, and nutrient content (Wright et al., 2004). High SLA values are generally recorded in resource-rich environments, whereas low values are recorded in resource-poor environments (Freschet et al., 2010; Pérez-Harguindeguy et al., 2013). LDMC quantifies leaf tissue density and nutrient retention capacity. High LDMC values indicate a preference for conserving nutrients. Species with high levels of LDMC have tough leaves that are highly resistant to hazards (Freschet et al., 2010; Lienin and Kleyer, 2012). Height and SLA are two key independent axes of plant ecological strategies (Westoby, 1998; Le Bagousse-Pinguet et al., 2017). SLA is a function of LDMC and leaf thickness, while LDMC may give more meaningful information (Pérez-Harguindeguy et al., 2013). For example, SLA misses the majority of its ecological explanation in species whose photosynthetic organs do not have the typical planar form, LDMC remains well defined (Hodgson et al., 2011).

The trait frequency distribution of plant communities was quantified using four moments: mean, variance, skewness, and kurtosis (Enquist et al., 2015). The mean is the average location of plant community traits as a result of environmental selection and species adaptation, whereas the variance reflects the range or dispersion of trait values within a local community (Gross et al., 2017). Skewness and kurtosis are the shape moments of the trait frequency distribution, which represent the rarity and evenness of trait values within local communities (Butterfield and Munson, 2016; Wieczynski et al., 2019). We calculated the community-weighted mean, variance, skewness, and kurtosis (all weighted by the relative aboveground biomass of species) of height, SLA, and LDMC for each community.


(1) $${}_{{\text{Mean}}_{\text{j}}=\sum_{i}^{n}P_{i}\,\text{Ti}}$$

(2) $$V\,a\,r\,i\,a\,n\,c\,e_{j}=\sum_{i}^{n}P_{i}\,{\left(\mathrm{Ti}-{\mathrm{Mean}}_{j}\right)}^{2}$$

(3) $$S\,k\,e\,w\,n\,e\,s\,s_{j}=\sum_{i}^{n}{\frac{P_{i}\,\left(\mathrm{Ti}-{\mathrm{Mean}}_{j}\right)}{V\,a\,r\,i\,a\,n\,c\,e_{j}^{3/2}}}^{3}$$

(4) $$K\,u\,r\,t\,o\,s\,i\,s_{j}=\sum_{i}^{n}\frac{P_{i}\,{\left(\mathrm{Ti}-{\mathrm{Mean}}_{j}\right)}^{4}}{V\,a\,r\,i\,a\,n\,c\,e_{j}^{2}}$$

where *P*_*i*_ and *T*_*i*_ are the relative aboveground biomass and the trait value of species *i*, respectively, in community *j* and *n* is the total number of species within community *j*. For each community, the sum of the relative aboveground biomass is equal to 100%.

### Null Models

In this study, null model was used to identify the relative dominance of deterministic and stochastic processes (Swenson, 2014). All species occurring in 80 plots of each region were regarded as species pools for that region. Then, we randomly shuffled the functional trait values using these species pools and generated 999 randomized communities. Subsequently, the standardized effect size (SES) of each trait moment calculated as the difference between the observed value and the mean value of the null communities divided by the standard deviation of value of the null communities:


(5) $$\text{SES}=\frac{\left(p\,a\,r\,a\,m\,e\,t\,e\,r_{o\,b\,s\,e\,r\,v\,e\,d}-m\,e\,a\,n\,\left(p\,a\,r\,a\,m\,e\,t\,e\,r_{n\,u\,l\,l}\right)\right)}{s\,t\,a\,n\,d\,a\,r\,d\,d\,e\,v\,i\,a\,t\,i\,o\,n\,\left(p\,a\,r\,a\,m\,a\,t\,e\,r\,n_{u\,l\,l}\right)}.$$

Due to the non-normality of the variables, the Wilcoxon test was conducted to test the significant deviations of the observed parameters (i.e., mean, variance, skewness, and kurtosis) from null expectations (SES = 0). Community assembly was considered non-random if SES was significantly different from 0 (Kraft and Ackerly, 2010).

Community-weighted variance (CWV) is equal to the functional dispersion defined by Rao’s quadratic entropy estimated using Euclidian dissimilarities (Rao, 1982; De Bello et al., 2009). Therefore, the SES values of the variances in height, SLA, and LDMC frequency distributions were used to further test the relative strength of different deterministic processes in regulating grassland community assembly. The SES of a CWV less than 0 indicates trait convergence among coexisting species (Blanchard et al., 2019), whereas an SES value greater than 0 indicates trait divergence patterns (Bernard-Verdier et al., 2012). Trait divergence and convergence patterns may result from multiple community assembly processes, such as abiotic filtering and competitive and facilitative interactions (Moeller, 2004; Grime, 2006; Cornwell and Ackerly, 2009; Mayfield and Levine, 2010). To avoid uncertainties in the accepted usage of the term “assembly process,” the trait-convergence and trait-divergence assembly processes were adopted in this study following the method of (Pillar et al., 2009).

### Statistical Analysis

Four moments (mean, variance, skewness, and kurtosis) of trait distribution and seven environmental variables (*T_*MWQ*_*, *T*_*S*_,*P_*MWQ*_*, *P*_*S*_, *S*_*TN*_, *S*_*TP*_, and *S*_*pH*_) were used in the statistical analysis. Prior to the analysis, all the explanatory variables were standardized. The data for variance and kurtosis of the trait frequency distribution were log-transformed to improve non-normality. The explanatory variables were divided into temperature stress, water availability, and soil attributes. The Wilcoxon test was employed to explore the differences in environmental variables and the moments of trait distribution between the TP and the MP. General linear and quadratic regression models were used to evaluate the relationship between the moments of trait frequency distribution and abiotic variables. Akaike information criterion (AIC) values were used to determine the better-fitting model (with a 10-unit reduction in the AIC value).

Stepwise multiple regression was used to further examine the most important drivers of the moments of the trait frequency distribution. The quadratic terms of the explanatory variables were also included in the initial models to account for possible quadratic relationships. To prevent data overfitting, all variables were subjected to forward selection until *P* < 0.05 for all explanatory variables. Variables were removed according to the criterion of a variance inflation factor > 3 to further avoid strong collinearity among variables. Hierarchical partitioning was applied to explore the independent effect of each variable using an R package.

## Results

### Shift in Functional Trait Frequency Distribution Along Environmental Gradients

**FIGURE 2:**
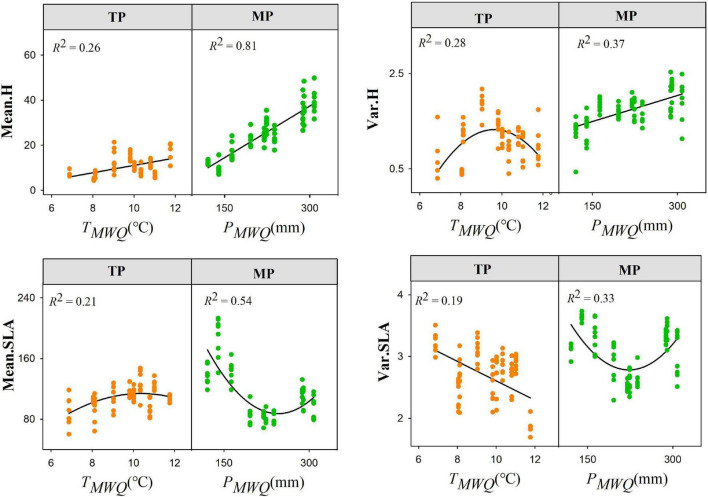
Shifts in the trait frequency distributions for height (H) and specific leaf area (SLA) within grassland communities along environmental gradients. Mean.H and Mean.SLA represent community-weighted mean of height and SLA, respectively; Var.H and Var.SLA represent community-weighted variance of height and SLA, respectively.

Both the mean and variance of the height, SLA, and LDMC frequency distributions were lower on the TP than on the MP (Supplementary Figure 2). However, there were no differences in skewness and kurtosis values. Furthermore, the responses of the four moments of height, SLA, and LDMC distributions to temperature stress and water availability differed between the TP and MP (Figure 2, Supplementary Figures 3–6, and Supplementary Tables 1–3). Both all moments of height and SLA distribution, and the skewness of LDMC distribution were more strongly related to *T_*MWQ*_* or*T_*S*_* in TP. In contrast, both all moments of height and LDMC distribution, skewness and kurtosis of SLA distribution showed stronger correlations with *P*_*MWQ*_ or*P_*S*_* in MP. Notably, the variance and kurtosis of LDMC distribution were only significantly related to *P*_*S*_ in TP, while the mean and variance of SLA were more strongly related to *T_*MWQ*_* in MP.

The mean height was positively related to *T_*MWQ*_* on the TP but showed a concave relationship on the MP (Supplementary Table 1). The mean height increased with *P*_*MWQ*_ on the MP but decreased on the TP. The variances in height had opposing responses to *T_*MWQ*_* between the TP and MP. Both the skewness and kurtosis of the SLA were significantly correlated with *P*_*MWQ*_ on the MP (*P* < 0.01) but only with *T_*MWQ*_* on the TP (*P* < 0.01) (Supplementary Table 2). The skewness of the LDMC distribution was positively related to *P*_*S*_ on the TP but negatively related to *P*_*S*_ on the MP (Supplementary Table 3). The shifts in the height and SLA distributions along the soil pH and nutrient gradients also varied between the TP and MP. For example, the mean and variance of both height and SLA increased with soil pH on the TP but not on the MP.

### Drivers of Trait Frequency Distribution

**FIGURE 3:**
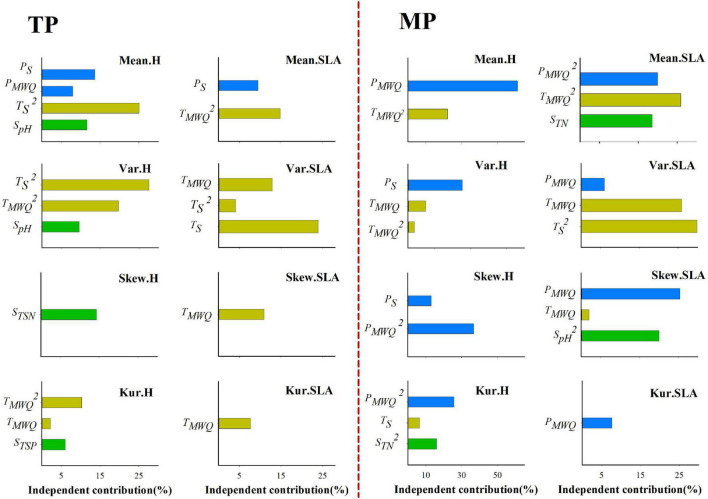
Independent influence of abiotic variables on the variations in mean, variance, skewness, and kurtosis of the height and SLA distributions within grassland communities. Skew.H and Skew.SLA represent community-weighted skewness of height and SLA, respectively; Kur.H, and Kur.SLA represent community-weighted kurtosis of height and SLA, respectively.

Stepwise multiple regression results showed that the four moments of the height, SLA, and LDMC frequency distributions are dependent on climatic factors on both the TP and MP (Supplementary Tables 4, 5). However, the relative influence of water- and temperature-related factors varied between the TP and MP. On the TP, *T*_*S*_ and *T_*MWQ*_* individually explained 0–47.30, 7.67–40.77, and 0–21.32% of the variation in the four moments of the height, SLA, and LDMC distributions, respectively (Figure 3 and Supplementary Figure 7), whereas water-related factors individually explained 0–13.69%, 0–9.46%, and 4.9–24.41% of the variation in those three trait distributions. On the MP, *P*_*S*_ and *P*_*MWQ*_ individually explained 30.28–61.21%, 6.02–25.40%, and 7.35–50.62% of the variations in the four moments of the height, SLA, and LDMC distributions, respectively (Figure 3 and Supplementary Figure 7), whereas *T*_*S*_ and *T_*MWQ*_* explained 0–22.08%, 0–62.04%, and 0–22.99% of the variations in those three trait distributions. These results indicate that both water- and temperature-related factors significantly influence the trait distributions on the TP and MP. However, on average, temperature-related factors determine the trait distributions on the TP, whereas water-related factors have greater influence on the trait distributions on the MP.

### Influence of Different Ecological Processes on Community Assembly

**FIGURE 4:**
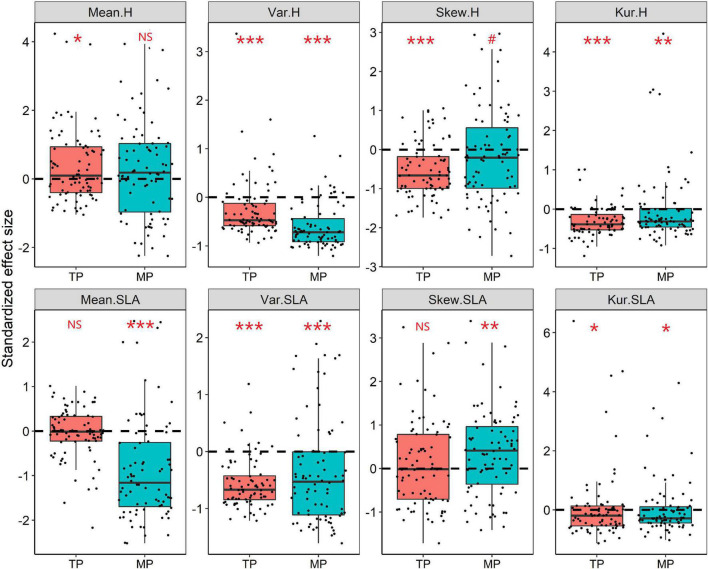
Standardized effect size (SES) values for the mean, variance, skewness, and kurtosis of the height and SLA distributions within grassland communities. The Wilcoxon test was conducted to test significant deviations within each observed trait metric from the null expectation (SES = 0), NS, *P* > 0.05; ^#^*P* < 0.1; *P < 0.05; **P < 0.01; ***P < 0.001. The black dashed line indicates the value of zero.

**FIGURE 5:**
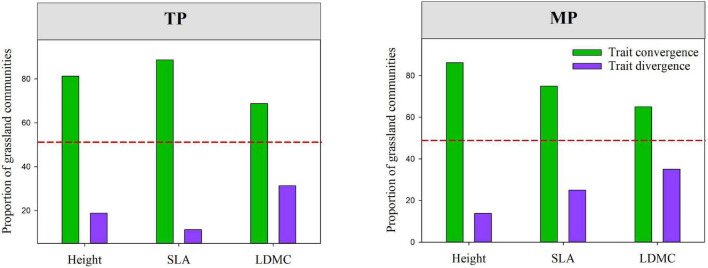
The relative prevalence of convergence and divergence within grassland communities among different regions, which were evaluated based on standardized effect values (SES) for the variance in height, SLA and, and leaf dry matter content (LDMC) distributions within grassland communities (SES.Var.H, SES.Var.SLA, and SES.Var.LDMC). The red dashed line indicates the 50% quantile. Convergence: SES < 0; divergence: SES > 0. SES. Var.H, SES.Var.SLA, and SES.Var.LDMC represent the standardized effect size values of community-weighted variance of height, SLA, and LDMC, respectively.

The results of the null model and Wilcoxon test demonstrated that almost all moments of height, SLA, and LDMC (except for mean and skewness of SLA) significantly deviated from their null expectations (SES = 0) on the TP (*P* < 0.05, Figure 4 and Supplementary Figure 8). Similarly, the variance and kurtosis of height significantly deviated from the null expectations on the MP. Furthermore, the mean SESs of the kurtosis of height, SLA, and LDMC distributions across all grassland communities on both the TP and MP were significantly less than 0 (Figure 4). The majority of grassland communities exhibited convergent frequency distributions for height (81.25%), SLA (88.75%), and LDMC (68.75%) on the TP (Figure 5). Convergent SLA, LDMC, and height distributions were also observed in 86.25, 65, and 75% of the grassland communities on the MP, respectively. The Wilcoxon test results further demonstrated lower observed variances of height, SLA, and LDMC than expected on both the TP and MP, when trait variance was evaluated as the mean value across all local grassland communities.

## Discussion

### Dominant Roles of Trait-Convergence Process in Driving Community Assembly

To elucidate the relative contributions of deterministic and stochastic processes in grassland community assembly across the TP and MP, we determined the differences between the observed and expected values for dominance, dispersion, rarity, and evenness of trait distributions. Our results demonstrated that almost all moments of the height, SLA, and LDMC frequency distributions significantly deviated from the null expectations, suggesting the dominance of deterministic processes (Kraft and Ackerly, 2010; Liu et al., 2020). More importantly, our results further demonstrated that trait convergence was more prevalent than trait divergence across grassland communities of the TP and MP, implying the widespread coexistence of functionally similar species within the grassland community. This indicates that trait-convergence processes, such as abiotic filtering or weaker competitive exclusion, govern grassland community assembly (Grime, 2006; Mayfield and Levine, 2010; Backhaus et al., 2021). Previous studies on forests have reported the prevalence of trait divergence in tree assemblages (Luo et al., 2019, 2021), while study on desert steppe observed a convergent trait frequency distribution (Wang X. et al., 2021). The interpretation for those discrepancy between forest and grassland might be that, harsh grassland environment leads to a stronger abiotic filtering or weaker competitive exclusion, which reduce the range of trait values (convergence; De Bello et al., 2009; Kraft and Ackerly, 2010), while limiting similarity dominates in weaker stressful forest, thereby results in a high trait diversity (divergence; Mayfield and Levine, 2010). This supports the viewpoints of environment stress governs the relative roles of different assembly processes, which resulting in different trait distribution patterns (Enquist et al., 2015; Lhotsky et al., 2016).

The mean SESs of the kurtosis of height, SLA, and LDMC distributions across all communities of the TP and MP were less than 0. This implies that the trait-convergence assembly process decreased the functional dispersion but increased the evenness of the trait frequency distribution. Functional traits can regulate species abundance by influencing fitness and performance (McGill et al., 2006; Webb et al., 2010). Trait-convergence processes, such as abiotic filtering or weaker competitive exclusion, maintain the species coexistence and filter out species with lower resistance and competitiveness, thereby reducing functional dispersion (Mayfield and Levine, 2010; Kraft et al., 2015; Šímová et al., 2015). However, species that enter the community will have relatively high and uniform fitness (Grime, 2006; Cornwell and Ackerly, 2009), which may decrease the rarity of traits.

### Response of Trait Distribution to Abiotic Stressors Between Tibetan Plateau and Mongolian Plateau

Habitat conditions may directly influence the trait distribution of plant communities (Schöb et al., 2013). Temperature and drought stresses are likely the most important macrofilters for grassland community assembly on the TP and MP, respectively (Zhang et al., 2020). Therefore, the observed shifts in different key features of trait distribution along these abiotic stress gradients can be linked to community assembly processes and how communities respond to climate change (Enquist et al., 2015). In line with the findings for global drylands (Le Bagousse-Pinguet et al., 2017), this study revealed significant shifts in four moments of the height, SLA, and LDMC frequency distributions along abiotic gradients on both the TP and MP. All evidence indicates that increasing abiotic stress may lead to different shapes of the trait frequency distributions, which in turn affects grassland community assembly (Kraft et al., 2015).

**FIGURE 6:**
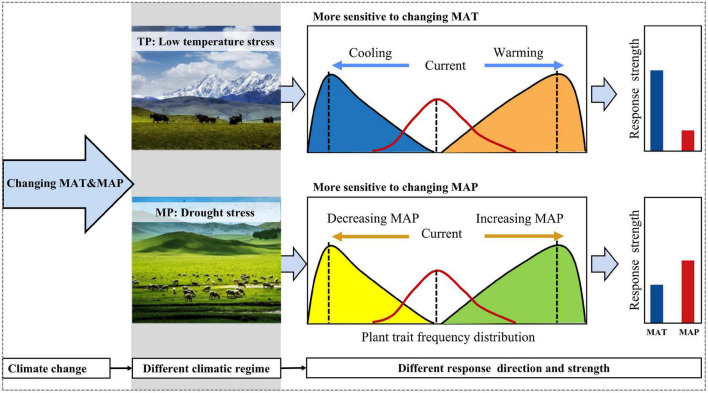
A theoretical framework for the different responses of community assembly to climate change. The specifics of the climatic regimes will govern the direction and strength of the responses to changes in climate. MAP, mean annual precipitation; MAT, mean annual temperature.

However, the responses of the four moments of height, SLA, and LDMC frequency distributions to abiotic stressors differed between the TP and MP. The mean and variance of the community height data may increase along the water availability gradient on the MP but decrease on the TP because hydraulic constraints are more prevalent on the MP than on the TP (Koch et al., 2004). Lower temperatures substantially reduced plant height on the TP, indicating that low-temperature stress strongly limits plant growth in alpine grasslands. In contrast, plant height increased at the two extremes of the temperature gradient across the MP because of the U-shaped relationship of summer precipitation with temperature, which resulted in lighter drought stress at the two extremes of the temperature gradient. Given that the temperature on the TP was lower than that on the MP (Zhang et al., 2020), these results suggest that species strategy responses to different stressors depend on the environmental regime (Muscarella et al., 2016; Crous et al., 2018). The variance of height and SLA were more strongly related to temperature-related factors on the TP but to water availability on the MP. The kurtosis of height and SLA responded prominently to low-temperature stress on the TP but had a more powerful response to drought stress on the MP. These results demonstrate that low-temperature and drought stresses determine the functional dispersion and evenness of grassland communities on the TP and MP, respectively. Furthermore, the skewness of the LDMC frequency distribution was positively (negatively) related to precipitation seasonality on the TP (MP). These results suggest that climatic regimes may mediate community assembly responses to abiotic stressors and even climate change (Figure 6).

### Different Abiotic Stressors Driving Community Assembly Processes on Tibetan Plateau and Mongolian Plateau

Temperature, precipitation, and their seasonality exerted the greatest individual effects on the four key moments of the height, SLA, and LDMC frequency distributions on the TP and MP, which demonstrates that climate is the dominant filter driving the trait frequency distribution (Wieczynski et al., 2019). However, we observed that soil pH and nutrient content individually explained 6.01–14.32% and 6.58–23.46% of the variation in some moments of height, SLA, and LDMC, respectively. Furthermore, these soil variables were highly correlated with climatic factors. Previous studies have reported that climate and soil interact to affect trait distributions (Simpson et al., 2016; Le Bagousse-Pinguet et al., 2017). Therefore, local environmental factors, such as soil pH and nutrient availability, also play an important role in influencing the trait frequency distribution in grassland communities (Le Bagousse-Pinguet et al., 2017; Umaña et al., 2021).

Our results further showed that temperature-related factors exerted a more individual effect on the height and SLA distribution of the TP, confirming the dominant role of temperature as an abiotic filter. The skewness of the LDMC distribution was more strongly influenced by temperature on the TP, whereas the variance and kurtosis were mainly determined by drought stressors. This suggests that drought stress plays an important role in shaping the trait distribution on the TP and that multiple leaf trait strategies respond to similar abiotic stresses. The largest fraction of the variations in height, SLA, and LDMC distributions was driven by water-related factors on the MP, which implies that water availability determines the distribution of plant traits within MP communities, which is consistent with the earlier findings (Butterfield and Munson, 2016). This result suggests that divergent abiotic drivers shape functional trait frequency distributions within grassland communities among different biogeographic regions.

We demonstrated that divergent abiotic stressors regulate the strength of trait convergence and divergence on the TP and MP, respectively, which partly supports the stress-dominance hypothesis (Coyle et al., 2014; Lhotsky et al., 2016). The SESs of the variance in height and SLA were mainly affected by temperature-related variables on the TP (Supplementary Figure 5). On the TP, community height was more convergent and less divergent at both ends of the temperature gradient, but community SLA tended to converge at moderate levels of temperature stress. This evidence supports the hypothesis that different traits vary substantially in their roles in trait convergence and divergence in community assembly (Ding et al., 2019). In contrast, water availability plays a key role in shaping the relative strength of the trait convergence and divergence on the MP. Community height became more convergent in more drought-prone habitats, but community SLA tended to exhibit trait convergence at moderate levels of drought stress. Community LDMC became more convergent in the higher precipitation seasonality region on the TP, while it tended to exhibit trait convergence at both ends of the drought gradient on the MP. These findings provide robust evidence that different abiotic stressors determine the relative importance of abiotic filtering and biotic interactions in the grassland community assembly on the TP and MP. Overall, our findings suggest that the effect of different aspects of future climate change, such as climate warming and changing precipitation patterns, on community assembly is dependent on regional climatic regimes, especially the specific limiting factor.

## Conclusion

Our results demonstrated that trait-convergence assembly processes, such as abiotic filtering or weaker competitive exclusion, as the dominant determinant of species coexistence in harsh environment of TP and MP, resulting in widespread coexistence of functionally similar species. However, different abiotic stressors regulated the community assembly of TP and MP. Low temperature stress acted as a strong filter determining the functional structure of alpine grassland in TP, while drought stress governed the temperate grassland assembly in MP. Our findings provide empirical evidence that regional climatic regimes govern grassland community assembly respond to environment stress. Community assembly may respond more strongly to future climate warming on the TP because of the coinciding temperature limitations and more rapid warming scenarios.

## Data Availability Statement

The original contributions presented in the study are included in the article/Supplementary Material, further inquiries can be directed to the corresponding author/s.

## Author Contributions

ML and NH conceived, designed the project, and led the data collection effort. JW, LX, ML, and PY participated in the transect investigation. JW performed the analyses and wrote the manuscript. CL helped with the data analysis. ML revised the manuscript. All authors discussed the results and contributed significantly to the final manuscript.

## Conflict of Interest

The authors declare that the research was conducted in the absence of any commercial or financial relationships that could be construed as a potential conflict of interest.

## Publisher’s Note

All claims expressed in this article are solely those of the authors and do not necessarily represent those of their affiliated organizations, or those of the publisher, the editors and the reviewers. Any product that may be evaluated in this article, or claim that may be made by its manufacturer, is not guaranteed or endorsed by the publisher.

## Footnotes

1
http://www.worldclim.org
