# Supplementary material for: Divergent Abiotic Stressors Drive Grassland Community Assembly of Tibet and Mongolia Plateau
Source: Front Plant Sci. 2022 Jan 3;12:715730. doi: 10.3389/fpls.2021.715730 (PMC8761913; doi:10.3389/fpls.2021.715730)
Supplement: Supplementary file 1 [file Data_Sheet_1.doc]

**Table S1** Relationships between four important parameters of frequency distribution for plant height in communities and environmental variables in grasslands of Tibet Plateau (TP) and Monglia Plateau (MP). *S_TN_*, soil total nitrogen content; *S_TP_*, soil total phosphorus; *S_pH_*, Soil pH; *T_MWQ_*, mean temperature of warmest quarter; *T_S_*, temperature seasonality; *P_MWQ_*, precipitation of wettest quarter; *P_S_*, precipitation seasonality. HS, hump-shaped relationship; US, U-shaped relationship; NS , *P* ＞ 0.05. Only when *P* < 0.05, slope and *R*^2^ were reported.

| **Moments** | **Variable** | **TP** | | |  | **MP** | | |
| --- | --- | --- | --- | --- | --- | --- | --- | --- |
|  |  | *R*^2^ | Slope | *P* |  | *R*^2^ | Slope | *P* |
| **Mean** | *S_pH_* | 0.14 | 0.39 | < 0.001 |  |  |  | NS |
|  | *S_TN_* | 0.28 | -0.54 | < 0.0001 |  | 0.08 | 0.3 | < 0.01 |
|  | *S_TP_* |  |  | NS |  |  |  | NS |
|  | *T_S_* | 0.32 | US | < 0.0001 |  | 0.57 | -0.76 | < 0.0001 |
|  | *T_MWQ_* | 0.26 | 0.52 | < 0.0001 |  | 0.42 | US | < 0.0001 |
|  | *P_MWQ_* | 0.09 | -0.32 | < 0.01 |  | 0.81 | 0.9 | < 0.0001 |
|  | *P_S_* | 0.08 | 0.31 | < 0.01 |  | 0.51 | 0.72 | < 0.0001 |
| **Variance** | *S_pH_* | 0.11 | 0.35 | < 0.01 |  |  |  | NS |
|  | *S_TN_* |  |  | NS |  | 0.07 | 0.28 | < 0.05 |
|  | *S_TP_* |  |  | NS |  |  |  | NS |
|  | *T_TS_* | 0.26 | US |  |  | 0.29 | -0.55 | < 0.0001 |
|  | *T_MWQ_* | 0.28 | HS |  |  | 0.13 | US | < 0.01 |
|  | *P_MWQ_* |  |  | NS |  | 0.37 | 0.61 | < 0.0001 |
|  | *P_PS_* |  |  |  |  | 0.32 | 0.57 | < 0.0001 |
| **Skewness** | *S_pH_* | 0.06 | -0.26 | < 0.05 |  | 0.05 | 0.25 | < 0.05 |
|  | *S_TN_* | 0.14 | 0.39 | < 0.001 |  | 0.46 | US | < 0.0001 |
|  | *S_TP_* |  |  | NS |  | 0.11 | -0.34 | < 0.01 |
|  | *T_S_* |  |  | NS |  | 0.15 | 0.4 | < 0.0001 |
|  | *T_MWQ_* |  |  | NS |  | 0.13 | 0.37 | < 0.0001 |
|  | *P_MWQ_* | 0.06 | 0.27 | < 0.05 |  | 0.49 | US | < 0.0001 |
|  | *P_S_* |  |  | NS |  | 0.21 | US | < 0.0001 |
| **Kurtosis** | *S_pH_* |  |  | NS |  |  |  | NS |
|  | *S_TN_* |  |  | NS |  | 0.46 | US | < 0.0001 |
|  | *S_TP_* | 0.06 | 0.26 | < 0.05 |  | 0.04 | -0.24 | < 0.05 |
|  | *T_S_* |  |  | NS |  | 0.05 | 0.24 | < 0.05 |
|  | *T_MWQ_* | 0.11 | US | < 0.01 |  | 0.09 | 0.32 | < 0.01 |
|  | *P_MWQ_* |  |  | NS |  | 0.41 | US | < 0.0001 |
|  | *P_S_* |  |  | NS |  | 0.21 | US | < 0.0001 |

**Table S2** Relationship between four important parameters of frequency distribution for specific leaf area (SLA) in communities and environmental variables in grasslands of Tibet Plateau (TP) and Mongolia Plateau (MP). *S_TN_*, soil total nitrogen content; *S_TP_*, soil total phosphorus; *S_pH_*, Soil pH; *T_MWQ_*, mean temperature of warmest quarter; *T_S_*, temperature seasonality; *P_MWQ_*, precipitation of wettest quarter; *P_S_*, precipitation seasonality. HS, hump-shaped relationship; US, U-shaped relationship; NS , *P* ＞ 0.05. Only when *P* < 0.05, slope and *R*^2^ were reported.

|  | Variable | TP | | |  | MP | | |
| --- | --- | --- | --- | --- | --- | --- | --- | --- |
|  |  | *R*^2^ | Slope | *P* |  | *R*^2^ | Slope | *P* |
| **Mean** | *S_pH_* | 0.05 | 0.25 | < 0.05 |  | 0.2 | HS | < 0.0001 |
|  | *S_TN_* |  |  | NS |  | 0.27 | -0.53 | < 0.0001 |
|  | *S_TP_* |  |  | NS |  | 0.16 | -0.4 | < 0.0001 |
|  | *T_S_* |  |  | NS |  | 0.64 | US | < 0.0001 |
|  | *T_MWQ_* | 0.21 | HS | < 0.0001 |  | 0.64 | HS | < 0.0001 |
|  | *P_MWQ_* |  |  | NS |  | 0.54 | US |  |
|  | *P_S_* | 0.09 | 0.32 | < 0.01 |  |  | NS |  |
| **Variance** | *S_pH_* | 0.1 | –0.34 | < 0.01 |  |  |  | NS |
|  | *S_TN_* | 0.26 | 0.52 | < 0.0001 |  | 0.18 | -0.44 | < 0.0001 |
|  | *S_TP_* |  |  | NS |  | 0.14 | -0.39 | < 0.001 |
|  | *T_S_* | 0.34 | US | < 0.0001 |  | 0.53 | US | < 0.0001 |
|  | *T_MWQ_* | 0.19 | -0.45 | < 0.0001 |  | 0.59 | US | < 0.0001 |
|  | *P_MWQ_* | 0.21 | 0.47 | < 0.0001 |  | 0.33 | US | < 0.0001 |
|  | *P_S_* | 0.06 | -0.27 | < 0.05 |  | 0.11 | 0.35 | < 0.01 |
| **Skewness** | *S_pH_* | 0.04 | -0.23 | < 0.05 |  | 0.3 | US | < 0.001 |
|  | *S_TN_* | 0.05 | 0.25 | < 0.05 |  |  |  | NS |
|  | *S_TP_* |  |  | NS |  |  |  | NS |
|  | *T_S_* |  |  | NS |  | 0.1 | -0.34 | < 0.001 |
|  | *T_MWQ_* | 0.11 | -0.35 | < 0.01 |  | 0.17 | US | < 0.0001 |
|  | *P_MWQ_* |  |  | NS |  | 0.35 | 0.6 | < 0.0001 |
|  | *P_S_* |  |  | NS |  | 0.19 | 0.45 | < 0.0001 |
| **Kurtosis** | *S_pH_* | 0.04 | -0.23 | < 0.05 |  |  |  | NS |
|  | *S_TN_* |  | NS |  |  |  |  | NS |
|  | *S_TP_* |  | NS |  |  |  |  | NS |
|  | *T_S_* |  | NS |  |  |  |  | NS |
|  | *T_MWQ_* | 0.08 | -0.3 | < 0.01 |  |  |  | NS |
|  | *P_MWQ_* |  |  | NS |  | 0.08 | 0.3 | < 0.01 |
|  | *P_S_* |  |  | NS |  |  |  | NS |

**Table S3** Relationship between four important parameters of frequency distribution for leaf fry matter content (LDMC) in communities and environmental variables in grasslands of Tibet Plateau (TP) and Mongolia Plateau (MP). *S_TN_*, soil total nitrogen content; *S_TP_*, soil total phosphorus; *S_pH_*, Soil pH; *T_MWQ_*, mean temperature of warmest quarter; *T_S_*, temperature seasonality; *P_MWQ_*, precipitation of wettest quarter; *P_S_*, precipitation seasonality. HS, hump-shaped relationship; US, U-shaped relationship; NS , *P* ＞ 0.05. Only when *P* < 0.05, slope and *R*^2^ were reported.

|  |  | **TP** | | |  | **MP** | | |
| --- | --- | --- | --- | --- | --- | --- | --- | --- |
|  | Variable | *R*^2^ | Slope | *P* |  | *R*^2^ | Slope | *P* |
| **Mean** | *S_pH_* | 0.13 | HS | < 0.01 |  | 0.25 | US | < 0.0001 |
|  | *S_TN_* | 0.06 | 0.27 | < 0.05 |  | 0.36 | 0.60 | < 0.0001 |
|  | *S_TP_* |  |  | NS |  | 0.21 | 0.47 | < 0.0001 |
|  | *T_S_* | 0.21 | HS | < 0.0001 |  | 0.41 | HS | < 0.0001 |
|  | *T_MWQ_* |  |  | NS |  | 0.62 | US | < 0.0001 |
|  | *P_MWQ_* | 0.22 | HS | < 0.0001 |  | 0.74 | HS | < 0.0001 |
|  | *P_S_* |  |  | NS |  |  |  | NS |
| **Variance** | *S_pH_* |  |  | NS |  | 0.21 | -0.52 | < 0.0001 |
|  | *S_TN_* |  |  | NS |  |  |  | NS |
|  | *S_TP_* |  |  | NS |  |  |  | NS |
|  | *T_S_* |  |  | NS |  |  |  | NS |
|  | *T_MWQ_* |  |  | NS |  | 0.10 | -0.33 | < 0.001 |
|  | *P_MWQ_* |  |  | NS |  | 0.24 | HS | < 0.0001 |
|  | *P_S_* | 0.24 | -0.50 | < 0.0001 |  | 0.10 | -0.34 | < 0.001 |
| **Skewness** | *S_pH_* | 0.15 | 0.40 | < 0.001 |  | 0.16 | HS | < 0.001 |
|  | *S_TN_* | 0.28 | -0.54 | < 0.0001 |  | 0.16 | -0.42 | < 0.001 |
|  | *S_TP_* |  |  | NS |  | 0.08 | -0.31 | < 0.05 |
|  | *T_S_* | 0.13 | 0.36 | < 0.001 |  | 0.17 | 0.41 | < 0.001 |
|  | *T_MWQ_* | 0.20 | 0.45 | < 0.0001 |  | 0.19 | HS | < 0.0001 |
|  | *P_MWQ_* | 0.08 | -0.30 | < 0.01 |  | 0.53 | US | < 0.0001 |
|  | *P_S_* | 0.24 | 0.50 | < 0.0001 |  | 0.25 | -0.51 | < 0.0001 |
| **K****urtosis** | *S_pH_* |  |  | NS |  |  |  | NS |
|  | *S_TN_* |  |  | NS |  | 0.22 | -0.48 | < 0.0001 |
|  | *S_TP_* |  |  | NS |  | 0.10 | -0.34 | < 0.01 |
|  | *T_S_* |  |  | NS |  |  |  | NS |
|  | *T_MWQ_* |  |  | NS |  | 0.12 | 0.36 | < 0.001 |
|  | *P_MWQ_* | 0.05 | -0.22 | < 0.05 |  | 0.32 | US | < 0.0001 |
|  | *P_S_* |  |  | NS |  | 0.28 | US | < 0.0001 |

**Table S4** Best predictor for the frequency distribution of height, SLA and LDMC within grassland communities of Tibet Plateau (TP). *S_pH_^2^*, the quadratic terms of soil pH; *S_TN_^2^*, the quadratic terms of soil total nitrogen content; *S_TP_^2^*, the quadratic terms of soil total phosphorus content; *T_MWQ_^2^*, the quadratic terms of mean temperature of warmest quarter; *T_S_ ^2^*, the quadratic terms of temperature seasonality; *P_MWQ_^2^*, the quadratic terms of precipitation of wettest quarter; *P_S_ ^2^*, the quadratic terms of precipitation seasonality. The numbers in brackets indicate the standardized regression coefficient of each variable that retained in the final model; *P* < 0.1; *, *P* < 0.05; **, *P* < 0.01; ***, *P* < 0.001.

|  | **Moments** | **Variable retained in the model** | **Model *R*^2^** | **Model *P*** |
| --- | --- | --- | --- | --- |
| **Height** | **Mean** | *S_pH_*(0.32**),*T_S_*^2^(0.42***),*P_MWQ_*(-0.25*),P_S_(0.43**) | 0.561 | < 0.0001 |
|  | **Variance** | *S_pH_*(0.28**), T_S_^2^ (0.39***),*T_MWQ_*^2^(-0.34***) | 0.552 | < 0.0001 |
|  | **Skewness** | *S_TN_*(0.39***) | 0.143 | < 0.001 |
|  | **Kurtosis** | *S_TP_*(0.25*), *T_MWQ_*^2^ (0.24*), *T_MWQ_*^2^ (0.33***) | 0.153 | < 0.001 |
|  |  |  |  |  |
| **SLA** | **Mean** | *T_MWQ_*^2^ (-0.37***), *P_S_*(0.30**) | 0.223 | < 0.0001 |
|  | **Variance** | *T_S_* (-0.55***), *T_S_*^2^ (-0.22**), *T_MWQ_* (-0.24*) | 0.384 | < 0.0001 |
|  | **Skewness** | *T_MWQ_* (-0.35**) | 0.109 | < 0.01 |
|  | **Kurtosis** | *T_MWQ_* (-0.29**) | 0.077 | < 0.01 |
|  |  |  |  |  |
| **LDMC** | **Mean** | *T_S_* (-0.41***),*P_MWQ_*^2^(-0.79***) | 0.347 | < 0.0001 |
|  | **Variance** | *P_S_*(-0.50***) | 0.244 | < 0.0001 |
|  | **Skewness** | *T_S_*(0.26***), *T_MWQ_* (0.21*),PS(0.41***) | 0.372 | < 0.0001 |
|  | **Kurtosis** | *P_MWQ_* (0.22*) | 0.05 | < 0.05 |

**Table S5** Best predictor for the frequency distribution of height, SLA and LDMC within grassland communities of Mongolia Plateau (MP). The numbers in brackets indicate the standardized regression coefficient of each variable that retained in the final model; *P* < 0.1; *, *P* < 0.05; **, *P* < 0.01; ***, *P* < 0.001.

|  | **Moments** | **Variable retained in the model** | **Model *R*^2^** | **Model *P*** |
| --- | --- | --- | --- | --- |
| **Height** | **Mean** | *T_MWQ_*^2^ (0.23**), *P_MWQ_* (0.79***) | 0.829 | < 0.0001 |
|  | **Variance** | *T_MWQ_* (-0.34**), *T_MWQ_*^2^ (0.39**), *P_S_*(0.61***) | 0.415 | < 0.0001 |
|  | **Skewness** | *P_MWQ_*^2^(0.75***), *P_S_*(-0.43***) | 0.486 | < 0.0001 |
|  | **Kurtosis** | *S_TN_*^2^(0.42***),*T_S_*(0.27**), *P_MWQ_*^2^(0.51***) | 0.461 | < 0.0001 |
|  |  |  |  |  |
| **SLA** | **Mean** | *S_TN_* (-0.38***), *T_MWQ_*^2^ (-0.70***), *P_MWQ_*^2^ (0.42***) | 0.631 | < 0.0001 |
|  | **Variance** | *T_S_*^2^(0.52***), *T_MWQ_* (0.48***), *P_MWQ_* (-0.32***) | 0.668 | < 0.0001 |
|  | **Skewness** | *S_pH_*^2^(0.21***), *T_MWQ_* (-0.21*), *P_MWQ_* (0.44***) | 0.454 | < 0.0001 |
|  | **Kurtosis** | *P_MWQ_* (0.30**) | 0.079 | < 0.01 |
|  |  |  |  |  |
| **LDMC** | **Mean** | *S_pH_*^2^ (0.15***), *T_S_* (-0.28***), *T_MWQ_*^2^(0.32***), *P_MWQ_*^2^ (-0.89***) | 0.793 | < 0.0001 |
|  | **Variance** | *S_pH_* (-0.46***),*P_MWQ_* (-0.21*) | 0.291 | < 0.0001 |
|  | **Skewness** | *S_pH_*^2^ (-0.16***), *P_MWQ_*^2^ (0.69***), *P_S_* (-0.50***) | 0.597 | < 0.0001 |
|  | **Kurtosis** | *P_MWQ_*^2^ (0.69***),*P_S_*(-0.35***) | 0.379 | < 0.0001 |


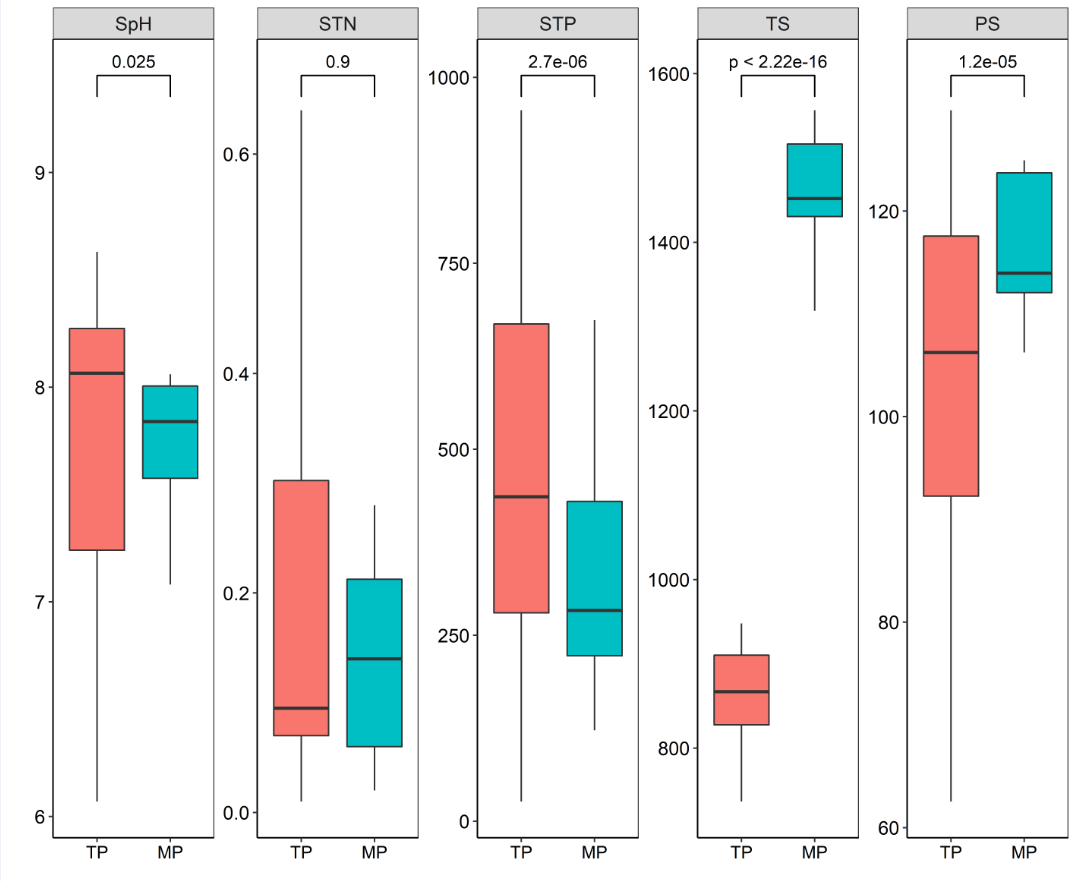


**Figure S1** Difference in climatic and soil conditions between the transects of Tibet Plateau (TP) and Mongolia Plateau (MP). Soil pH, pH; soil total nitrogen content, STN; soil total phosphorus content, STP; temperature seasonality, TS; precipitation seasonality, PS. Wilcoxon test was conducted to examine the significant difference in each soil attribute between TP and MP.


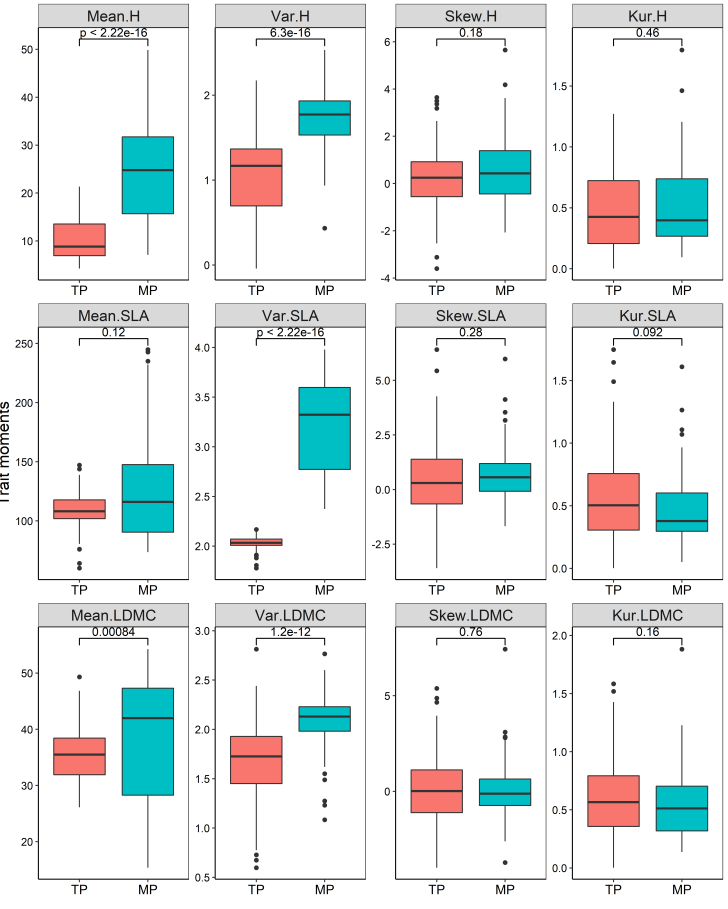


**Figure S2** Observed values of mean, variance, skewness, and kurtosis of height, SLA and LDMC frequency distribution within grassland communities of Tibet Plateau (TP) and Mongolia Plateau (MP). Mean.H, community-weighted mean of height; Var.H, community-weighted variance of height; Skew.H, community-weighted skewness of height; Kur.H, community-weighted kurtosis of height; Mean.SLA, community-weighted mean of speciﬁc leaf area; Var.SLA, community-weighted variance of speciﬁc leaf area; Skew.SLA, community-weighted skewness of speciﬁc leaf area; Kur.SLA, community-weighted kurtosis of speciﬁc leaf area; Mean.LDMC, community-weighted mean of leaf dry matter content; Var.LDMC, community-weighted variance of leaf dry matter content; Skew.LDMC, community-weighted skewness of leaf dry matter content; Kur.LDMC, community-weighted kurtosis of leaf dry matter content. Wilcoxon test was conducted to examine the significant difference in mean, variance, skewness, and kurtosis of three trait distribution between TP and MP.


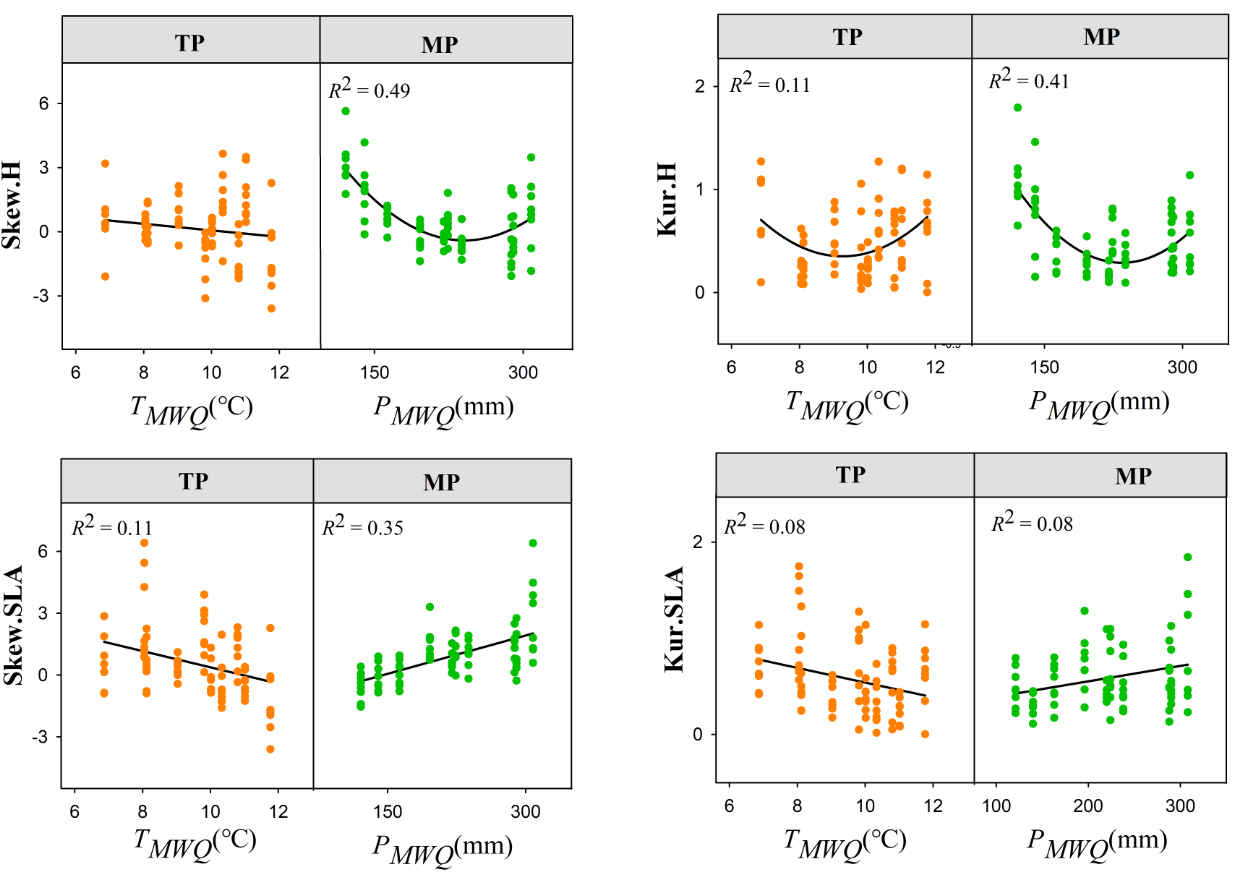


**Figure S3** Shifts in the observed values of skewness and kurtosis of height and SLA frequency distribution within grassland communities with changing temperature and precipitation gradient


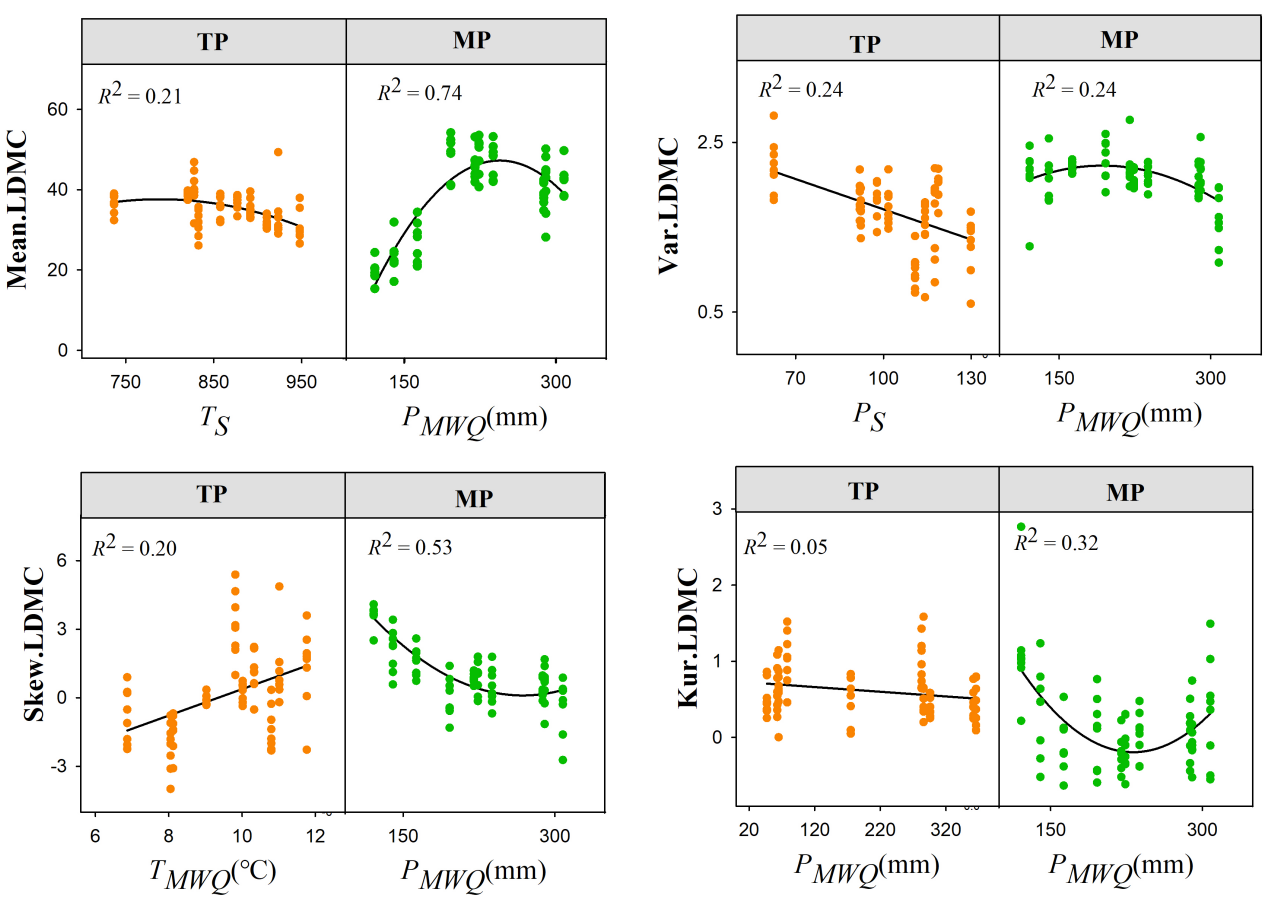


**Figure S4** Shifts in the observed values of mean, variance , skewness and kurtosis of LDMC frequency distribution within grassland communities with changing temperature and precipitation gradient


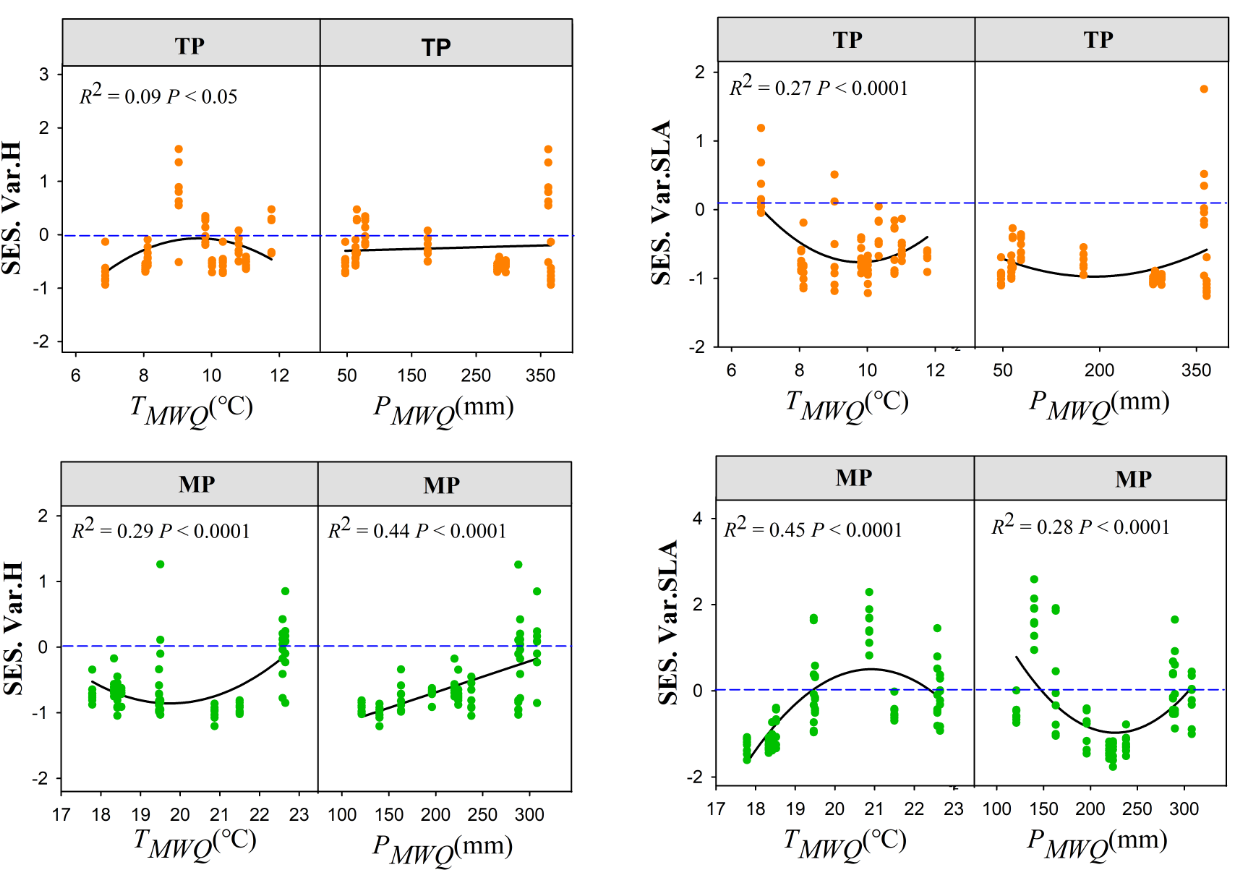


**Figure S5** Shifts in the standardised effect size values of variance for height and SLA frequency distribution with changing temperature and precipitation gradient


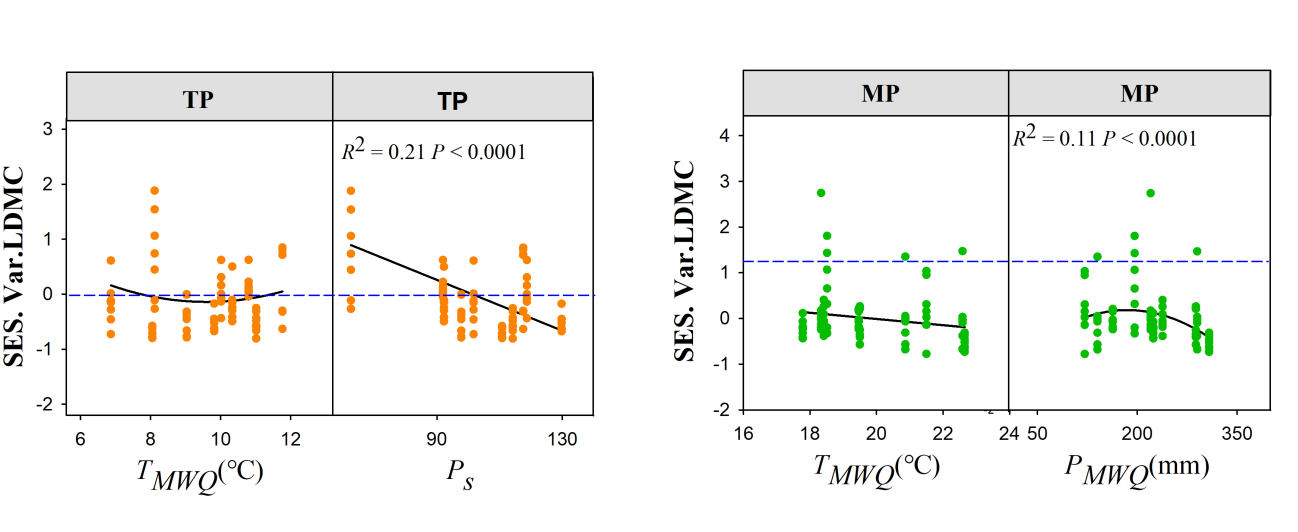


**Figure S6** Shifts in the standardised effect size values of variance for height and SLA frequency distribution with changing temperature and precipitation gradient


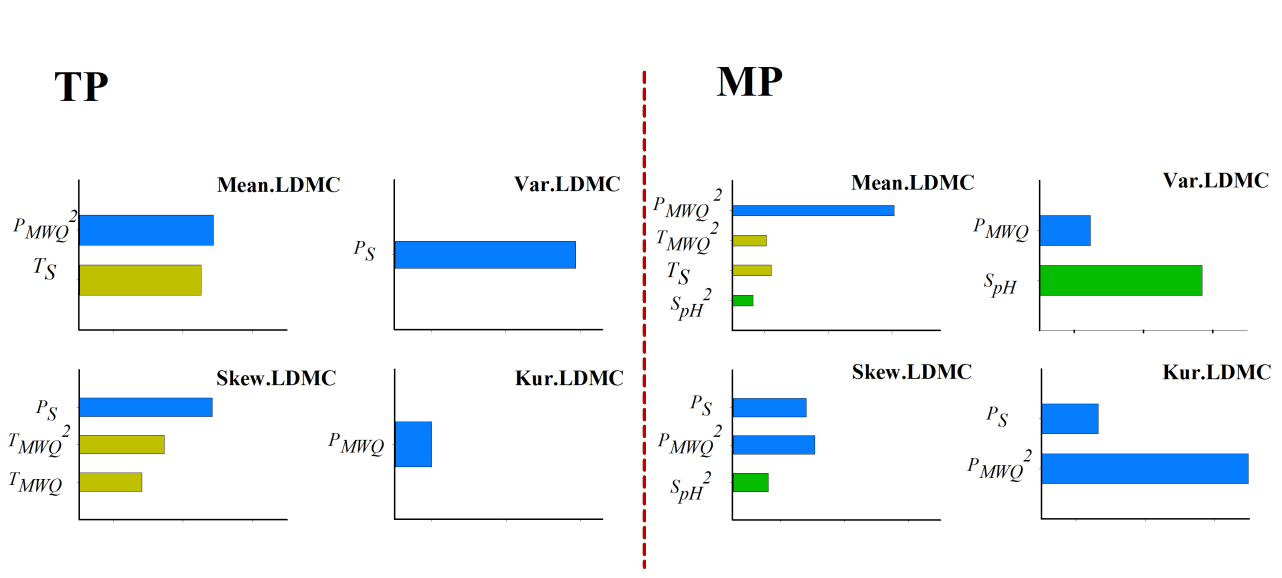


**Figure S7** Independent influence of abiotic variables on the variations in mean, variance, skewness, and kurtosis of the LDMC distributions within grassland communities.


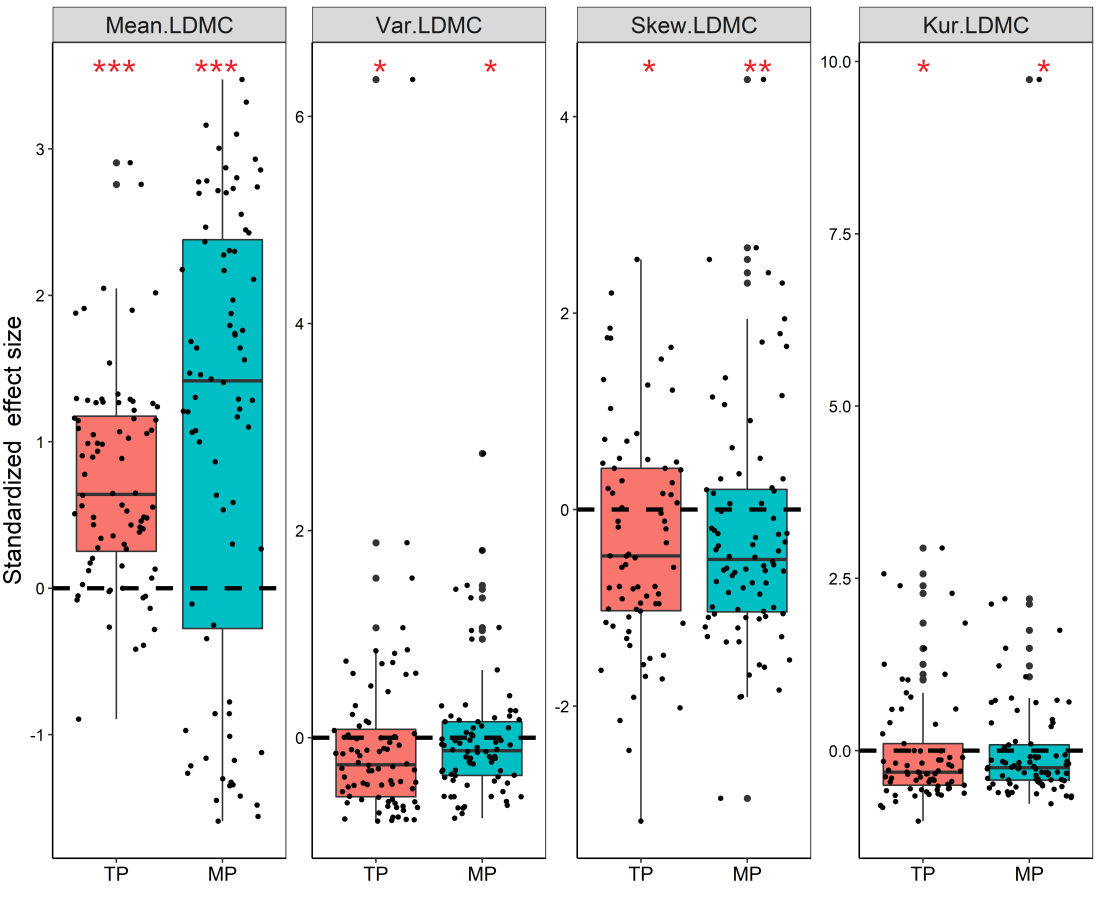


**Figure S8** Standardized effect size values (SES) for the mean, variance, skewness, and kurtosis of the LDMC distributions within grassland communities. A Wilcoxon test was conducted to test for significant deviations within each observed trait metric from the null expectation (SES = 0), *P* < 0.1; *, *P* < 0.05; **, *P* < 0.01; ***, *P* < 0.001. Black dashed line indicates a value of zero.
